# Hyaluronic acid–lipid binding

**DOI:** 10.1186/s13065-021-00763-0

**Published:** 2021-05-27

**Authors:** Anthony Ewurum, Abhishek Ashok Alur, Margaret Glenn, Abigail Schnepf, Douglas Borchman

**Affiliations:** 1 Department of Chemistry, University of Louisville, Louisville, KY 40296 USA; 2 Department of Ophthalmology and Visual Sciences, University of Louisville, 301 E Muhammad Ali Blvd., Louisville, KY 40202 USA

**Keywords:** Dye eye, Hyaluronan, Liquefaction, Nuclear magnetic resonance, Phospholipids, Vitreous humor

## Abstract

**Background:**

Phospholipid (PL)–hyaluronic acid (HA) interactions are relevant to aging-associated vitreous humor liquefaction, therapies for dry eye disease, skin-care products and synovial joint lubrication. Phosphatidyl choline–HA interactions have been well characterized. However, other major lipids found in tears, vitreous humor and synovial joints have not. The purpose of this study was to bridge this gap of knowledge.

**Methods:**

HA (1600 kDa) at 5 mg/mL, was mixed with various lipids ranging in concentration from 0.1 to 10 mg/mL in D_2_O. HA–PL binding was measured from the decrease in HA proton resonance intensity with binding using a nuclear magnetic resonance spectrometer.

**Results:**

Cholesterol weakly bound to HA, followed by monoglyceride and palmitoyl palmitate < phosphatidyl choline, phosphatidic acid and sphingomyelin. The maximum amount of PL bound was 14 ± 1 µmoles inferring a 1 to 1 molar ratio of bound PL to HA dimer. Monoglyceride and palmitoyl palmitate required two to three times more lipid to achieve 100% bound HA compared to PL.

**Conclusions:**

Physiological levels of HA, phosphatidyl choline and sphingomyelin would result in only 4% of the hydrophobic hydrogens of HA to be bound. HA–PL binding interactions could be important for therapeutic use of HA in eye drops in future studies to treat dry eye and to trap PL entering the VH to keep them from forming light scattering micelles. HA–lipid binding may also be relevant to the therapeutic effects of topical skin-care products. Both head group and hydrocarbon chain moieties influence HA–lipid interactions.

## Introduction

**Fig. 1:**
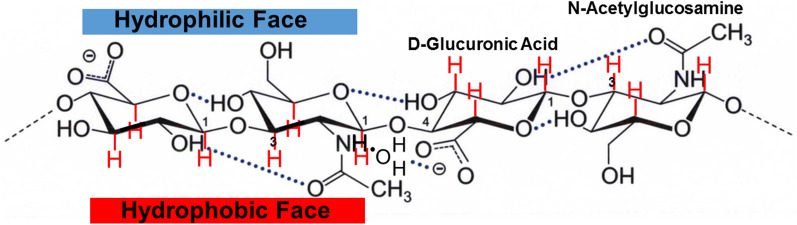
Hyaluronic acid polymeric structure. Protons in the hydrophobic face are in red

Hyaluronic acid (HA) is a polysaccharide with repeating units made up of glucuronic acid (GlcA), and an amino sugar, glucosamine (N-GlcNA), linked via a glycosidic bond (Fig. 1). It was first detected in 1934 in bovine vitreous humor (VH) [1]. The structure of HA was determined in 1950 [1–3]. The length of the HA chain varies by species and region. For example, rabbit VH has 2000–3000 kDa HA strands but bovine HA strands are shorter at 500–800 kDa [4]. Along the polysaccharide chains, hydrogen bonds cause the strand to twist, forming a helical ‘ribbon’ with hydrophobic and hydrophilic regions between the 1–3 and the 1–4 linkages [5, 6]. About half of the HA found in the human body is present in the skin [7], the remainder found in synovial fluid [8], the vitreous body [9] the umbilical cord [10] and places where friction occurs such as joints, tendons, sheaths, pleura, and the pericardium.

HA was first used to replace vitreous during an ocular surgery in the 1950’s [11]. Other applications of HA include: visco-supplementation in joints and restoration of synovial fluid, vocal cord therapy and sinus surgery, filling of facial wrinkles and depressed scars, and drug delivery [12]. Of importance to the current study, HA is also used in eye drops to treat dry eye [3, 13–16]

One disaccharide unit of HA can interact with 15 water molecules to increase its molecular weight up to 1000 fold [11, 17]. As discussed below, HA–phosphatidylcholine (PC) interactions have been well characterized, however, HA–phospholipid (PL) interactions involving other PLs have not. The intensity of the resonances of the methyl protons on the N-GlcNA decrease upon interacting with PLs due to resonance broadening and shielding. Furthermore, NMR data in addition to gel permeation chromatography and multi-angle laser-light scattering, suggest possible interactions between the PC tails and the hydrophobic patches of HA. In general, PC assembly involves assembly aggregation around HA strands, but HA molecular weight and calcium cations can affect this organization [18–21].

The historical importance of structural changes to HA induced by PLs in synovial fluids have been reviewed [5, 22]. It has been speculated that HA–lipid hydrophobic interactions in synovial fluids contribute to lubricating and protecting joint cavities. The importance of HA in relation to providing an elastic hydrostatic cushion and retaining water in the synovial fluid in the cavity of diarthrodial joints was recognized in 1953 [23]. HA is a major constituent of synovial fluid, 2 to 4 mg/mL [24]. PL are also major constituents of synovial fluid, 0.14 mg/mL [24]. PC is the major PL in synovial fluids at 61% of the PL. Sphingomyelin and cholesterol account for 19% and 15% of the lipids, respectively. Other lipids such as phosphatidyl ethanolamine, phosphatidyl inositol, cholesterol, monoglycerides, diglycerides, triglycerides, and free fatty acids are also present [24].

PLs have been found in human [25] and porcine VH [26]. The lipids in the anterior region of the VH come from the lens and the lipids in the posterior region of the VH come from the retina. Elevated levels of lipid are observed in the VH of humans with diabetes [26] and it is speculated that these lipids contribute to vitreous liquefaction [25, 26]. Liquefaction is the result of the collapse or contraction of the collagen/hyaluronan network, where collagen can be considered to act as the scaffold and HA fills the spaces in between [1, 27]. It has been speculated that lipids binding to HA, a major component of the VH other than water [6, 17, 27], could disrupt HA-collagen interactions leading to vitreous liquefaction and retinal detachment. The liquefaction of the VH, which is associated with aging, begins as early as the second decade of life and almost 50% of the VH is liquefied by the 8th and 9th decades of life [27]. The age-related changes that cause this liquefaction are not known at the molecular level. Free radicals, generated by metabolic processes or by photosensitized reactions, have been shown to induce depolymerization and conformational changes in HA [28]. Additionally, diabetic patients develop VH degeneration earlier in life. Liquefaction and thinning of the VH weaken the adhesion between the posterior VH cortex and the inner limiting membrane of the retina which can ultimately lead to posterior vitreous detachment, whereby the vitreous separates from the retina [1, 29].

HA–lipid binding could also be relevant to dry eye treatment, as HA is used with other therapeutics in eye drops to treat dry eye [3, 13, 16]. A thin layer of lipid coats the surface of tears and contributes to tear film stability [30–33]. The tear film lipid layer (TFLL) consists of PL [31] and (*O*-acyl)-ω-hydroxy fatty acids that partition at the interface region between the TFLL and tear aqueous [33]. Above the PL is the bulk lipid layer consisting of wax and cholesteryl esters over 17 molecules thick.

HA is often used in topical skin-care products [34]. It has been shown to stimulate collagen formation [35] and suppress sebum production [36]. Liposomes prepared from PL have been used to deliver HA by injection as a treatment for wrinkles [37]. Thus, these topics are relevant to the current study as HA–PL interactions are important to liposome encapsulation and HA-skin interactions. Wax and cholesteryl ester interactions with HA are important as they are the major components of skin sebum [38].

Only PC–HA binding has been qualitatively measured thus far. Other lipids such as phosphatidyl ethanolamine, sphingomyelin and cholesterol are present in the VH, and synovial fluid, and wax esters, and other PLs are present in the TFLL. As HA is used in eye drops as a therapy for dry eye, and PL–HA binding especially with diabetes, could contribute to vitreous liquefaction. The aim of the current study therefore, was to quantify PL–HA, cholesterol–HA and wax–HA binding.

## Materials and methods

### Materials

Polymeric HA sodium salt from Streptococcus equi [(α-ΔGlcU-(1 → 3)-GlcNAc)n] (MW = 1500 to 1800 kDa), D_2_O and palmitoyl sphingomyelin (pSM), dipalmitoyl phosphatidylcholine (DPPC), dipalmitoyl phosphatidic acid (DPPA), palmitoyl palmitate (PP), palmitoyl glyceride (PG) and cholesterol (Cho) were purchased from Sigma-Aldrich (St. Louis, MO).

### Sample preparation and binding assay

Stock solutions of HA and lipids were prepared in D_2_O at a concentration of 10 mg/mL. They were homogenized using a microprobe sonicator (Branson, Ultrasonics Co., Danbury, CT, USA) three times for 30 s with a 2-min rest period between sonication, at a probe setting of 4. Samples for the binding study were prepared with a final volume of 0.5 mL with a final HA concentration of 5 mg/mL (13.15 µmoles dimer). Samples were allowed to equilibrate for 30 h under argon gas (analyzed, ultra-pure; Welders Supply, Louisville, KY) to avoid oxidation. The samples were placed in nuclear magnetic resonance (NMR) tubes for analysis.

### NMR analysis

**Fig. 2:**
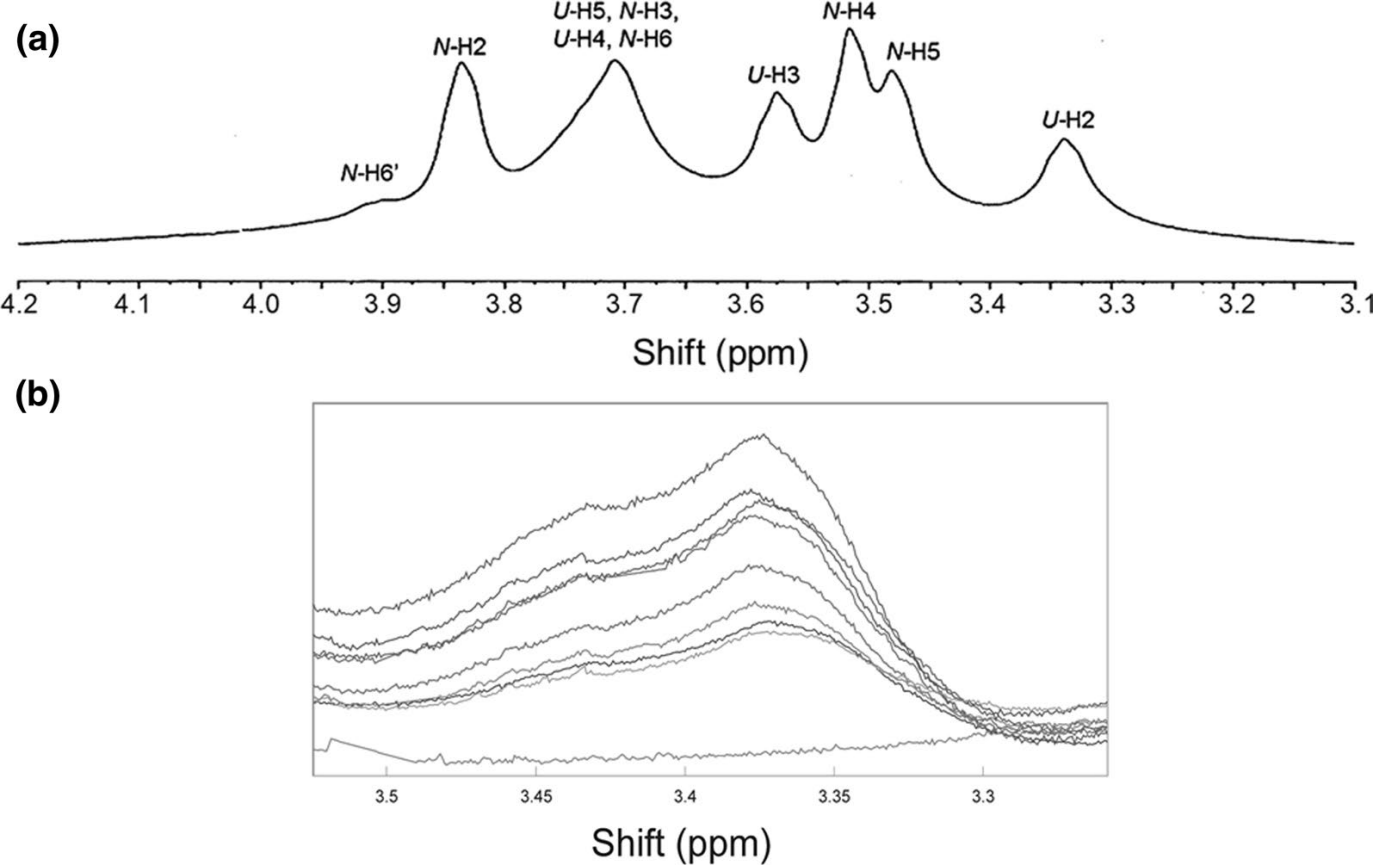
**a** A typical ^1^H-NMR spectrum of high molecular weight hyaluronan in D_2_O at 25 °C. U = d-glucuronic acid moiety, N = *N*-acetyl glucosamine moiety. **b** Effect of increasing dipalimitoyl phosphatidyl choline on the ^1^H-NMR spectra of hyaluronan. (Top to bottom) increasing concentrations of dipalimitoyl phosphatidyl choline as in Fig. 3a. The absolute intensities of the resonances of the HA–PL mixtures change from run to run due to instrumental tuning differences and the number of scans, so the data in Table 2 and in Fig. 3 were calculated and plotted relative to the intensity of the D_2_O resonance at 4.79 ppm

NMR spectra were obtained on a 700 MHz NMR spectrometer equipped with a 5 mm ^1^H{^13^C/^15^N} ^l3^C enhanced PFG cold probe (Palo Alto, CA). All ^l^H spectra were acquired with a minimum of 250 scans, 45° pulse width, and a relaxation delay of 1.000 s. All spectra were obtained at 25 °C unless stated otherwise. Spectra were manipulated and quantified using GRAMS/386 software (Galactic Industries, Salem, NH). A typical spectrum of HA is shown in Fig. 2. In some instances, such as with sphingomyelin, a sharp lipid band appeared and was removed using the ‘zap’ function. The intensity of the HA resonances was measured between 3.1 and 4.2 ppm. The absolute intensities of the resonances of the HA–PL mixtures change from run to run due to instrumental tuning differences and the number of scans, so the data in Table 2 and in the Figures were calculated relative to the intensity of the D_2_O resonance at 4.79 ppm. The following equation was used to measure the % decrease in HA resonance intensity, a lower level measure of the % lipid bound where I is the resonance intensity:

1 $$\left[ {\left( {\left( {{\text{I}}_{{{\text{HA}}}} /{\text{I}}_{{{\text{D2O}}}} } \right){\text{no lipid}} - \left( {{\text{I}}_{{{\text{HA}}}} /{\text{I}}_{{{\text{D2O}}}} } \right){\text{plus lipid}}} \right)/\left( {{\text{I}}_{{{\text{HA}}}} /{\text{I}}_{{{\text{D2O}}}} } \right){\text{no lipid}}} \right] \times {1}00/0.{6}$$

The constant 0.6 was calculated from 6 hydrophobic protons that bind to lipid/10 total protons in a HA dimer (Fig. 1). Data are presented ± the standard error of the mean.

## Results

**Fig. 3:**
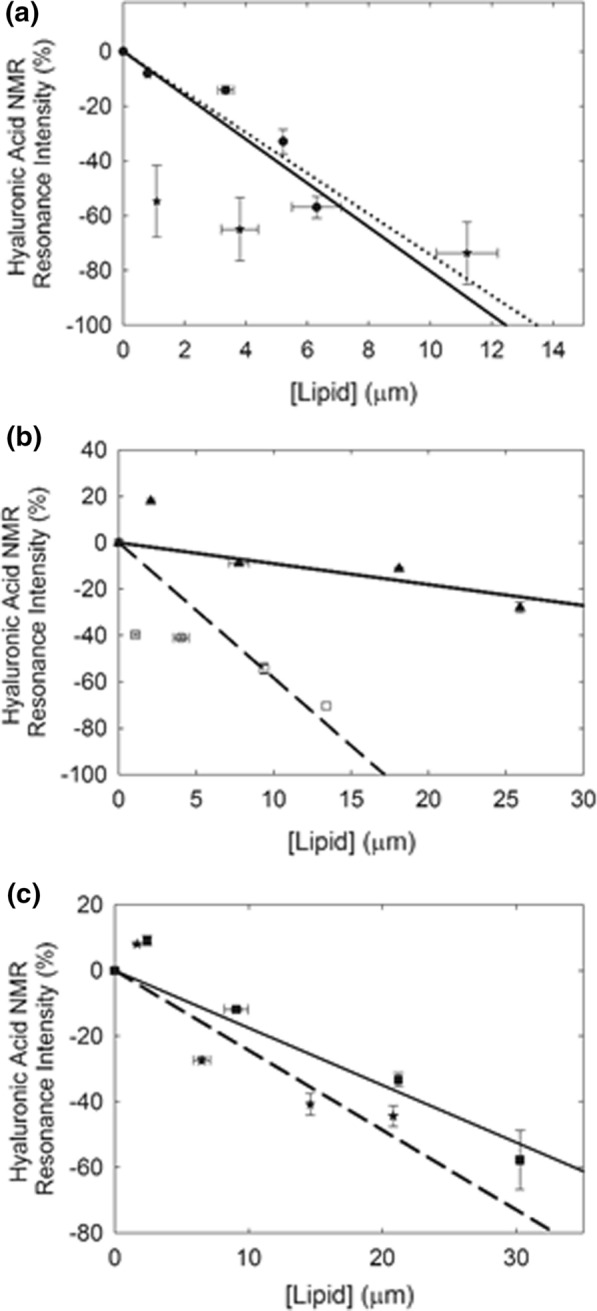
HA–lipid binding profiles. **a** Phospholipids with a choline headgroup () Phosphatidyl choline; () sphingomyelin. **b** () Cholesterol; () Phosphatidic acid. **c** Lipids found in the tear film lipid layer () the wax palmitoyl palmitate, () mono-glyceride. Lines are the linear regression fit

A typical ^1^H-NMR spectrum of high molecular weight HA is shown in Fig. 2a. The region of the ^1^H-NMR spectrum encompasses protons that are in the hydrophobic lipid-binding region and hydrophilic region of the glycerides (Fig. 1). When lipids bind to HA, the intensities of the proton resonances decrease (Fig. 3a, b) suggesting HA–lipid interactions broaden the resonance and shield protons from the magnetic field. The decrease in the HA intensity with increasing lipid concentration (Fig. 2b) is a lower level of HA–lipid binding since lipid could bind to HA and not decrease the intensity of the HA resonance. The absolute intensity changes from run to run due to instrumental tuning differences and number of scans so data in Table 1 and in Fig. 3 were calculated relative to the intensity of the D_2_O resonance at 4.7 ppm. Cho weakly binds to HA (Fig. 3b), followed by PG and PP < DPPC, and thereafter, PPA and pSM. Extrapolating the best-fit linear regression line to 100% bound, the maximum amount of phospholipid bound was 14 ± 1 µmoles, close to the value of 13.15 µmoles HA dimers in the assay (Fig. 3, Table 2). This indicates a 1 to 1 molar ratio of bound lipid to HA dimer. Two to three times more PG and the wax PP were however needed to reach 100% bound HA when compared to the other phospholipids, suggesting that they have a higher binding constant. The two choline containing phospholipids, DPPC and pSM both had a maximum binding ratio of 1:1 HA:PL (Table 2), however, pSM initially decreased the intensity of the HA resonances much more than DPPC (Fig. 3a). DPA without a choline head group but with the same acyl chains as DPPC both had a maximum binding ratio of 1:1 HA:PL, however, DPA initially decreased the intensity of the HA resonances much more than DPPC (Fig. 3a, b).

**Table 1:** ^1^H chemical shift assignments for hyaluronan

Proton number (see Fig. 1)	Experimental High-molecular weight polymer (ppm)	Literature^a^ Low-molecular weight polymer (ppm)	Literature^b^ HA_6_ (ppm)
*N*-acetyl glucosamine			
H1	4.55	4.55	4.55
H2	3.83	3.83	3.84
H3	^c^(3.71)	^c^3.73	3.71
H4	3.51	3.51	3.52
H5	3.48	3.47	3.48
H6	3.9	3.91	3.91
H6ʹ	^c^(3.71)	3.71	3.76
H-CH_3_	2.02	2.01	2.02
d-glucuronic acid			
H1	4.45	4.46	4.46
H2	3.33	3.34	3.34
H3	3.58	3.58	3.58
H4	^c^(3.71)	3.74	3.74
H5	^c^(3.71)	3.72	3.70

^a^ In D_2_O at 30 ° [39]

^b^In 10% D_2_O/H_2_O at 24 °C [40]

^c^Unresolved resonance

**Table 2:** Hyaluronan–lipid binding parameters from Fig. 3

Lipid	Slope (% bound/µmoles lipid)	X-axis intercept (µmoles)
Cholesterol	− 0.9	110 ± 42
Monoglyceride	− 1.8	57 ± 13
Palmitoyl palmitate	− 2.4	41 ± 12
Dipalmitoyl phosphatidic acid	− 5.8	17 ± 5
Palmitoyl sphingomyelin	− 8.0	13 ± 6
Dipalmityol phosphatidylcholine	− 7.4	13 ± 3

± 95% confidence interval

HA concentration was 13.15 µmoles in 0.5 mL D_2_O

## Discussion

The binding of PC to HA has been well characterized and reviewed [41]. The current study is unique in that HA–lipid interactions were measured with a range of lipid concentrations using a variety of lipids such as PC, sphingomyelin and wax. The lipid studied are relevant to vitreous liquefaction, tear film stability and lubrication of synovial joints. HA–cholesterol binding was relatively weak. A unique finding of our study is that HA does bind to waxes and cholesteryl esters, two lipids found in tears. The ^1^H-NMR lipid–HA binding assay presented in the current study is novel and could be used in future studies to quantify the binding characteristics of lipids to HA. The basis for the NMR assay is that when lipids interact with HA, the HA protons involved in the interaction spin slower, causing the resonances to become broader and less intense.

Our assay used a concentration of 5 mg/mL HA, close to the physiological value found in synovial fluids [22]. The molecular weight of HA does influence lipid–HA interactions [18–21]. The molecular weight of the HA used in our assay was between 1500 and 1800 kDa, similar to that found physiologically. PL titration of HA with different molecular weights has not been done. It is reasonable to speculate that small differences in the molecular weight of HA does not affect lipid binding as the hydrophobic face of the polymeric structure presented to the lipid for binding is the same for all molecular weight HA near to the MW of the HA used. By interpolating the binding from our plots in Fig. 3a, we calculate that physiological levels of HA, PC and sphingomyelin [22] would bind only 4% of the hydrophobic hydrogens of HA. Our data suggest that the cholesterol in synovial fluids would not influence HA at all. Whether HA–lipid hydrophobic interactions in synovial fluids contribute to lubricating and protective properties in joint cavities at a level of 4% binding has yet to be determined. HA–lipid hydrophobic interactions in vitro would be even less in arthritic joints in vivo considering that HA is degraded to low-molecular weight moieties that interact with lipid less than high molecular moieties of HA [18]. Perhaps higher levels of phospholipids, especially sphingomyelin, could lubricate and protect joint cavities therapeutically.

The adult VH HA concentration is approximately 0.2 to 0.3 mg/mL (~ 0.5 µmoles HA diamers) in the human VH depending on age [4]. One may question if there is enough PL in the VH to bind to HA to contribute to VH liquefaction. PL synthesis and types have been quantified in animals [2, 26, 42–47]. The concentration of PLs in the VH of cows, sheep, rabbits, rodents and dogs ranges approximately from 1 to 130 nmoles/mL [43, 45, 48, 49]. Based on our binding studies, at the highest concentration of PL in the VH of animals, assuming 1:1 binding, 24%, of the total HA would be expected to bind to PL. However, even less HA–PL binding would be expected in human VH since humans have a lower amount of PL in the VH ~ 3 nmoles/mL [49] compared to animals. Whether < 5% binding of PL to HA contributes to VH liquefaction has yet to be determined.

No significant differences between the concentrations of HA in the VH of diabetic and non-diabetic patients have been measured [48]. Therefore, with diabetes, only an increase in PL concentration in the VH could potentially increase HA–PL. Relatively higher levels of PLs and glucose in the VH of diabetic patients compared to VH from non-diabetics has been observed [25] and is believed to be caused by a decrease in the blood–retinal barrier with diabetes [50]. Quantitative studies to determine PL concentrations in the VH of diabetics are needed to determine if PL contributes to increased VH liquefaction in diabetic patients.

HA–PL binding could be important to trap PL entering the VH to keep them from forming light scattering micelles. Lack of Cho–HA binding could be why there is little Cho found in the VH [26] as it is likely to pass through the VH unimpeded.

Therapies for complicated retinal detachments often includes the removal of the VH (vitrectomy), and its replacement with an endotamponade such as perfluorocarbons, air, gases and silicone oils and hydrophilic, hydrogel-based systems such as hyaluronic acid, has been reviewed [51]. The strategy behind the tamponade is to stabilize the retina. HA tamponades such as HA offer the advantage over hydrophobic tamponades in that they do not leave a small amount of liquid at the opposite pole of the buoyancy vector wherein growth factors accumulate and promote pro-inflammatory processes [51]. Hydrophobic tamponades also lead to complications such as emulsification, cataract formation, and the need for revision surgery. HA used as a vitreous substitute provides optical, viscoelastic, and biocompatible properties. It would be interesting to see how lipid binding to HA, if it occurs over time, affects the biocompatible properties.

As discussed in the Introduction, HA–lipid binding could also be relevant to dry eye treatment as HA is used with other therapeutics in eye drops to treat dry eye [3, 13–16]. The current study indicates that HA in eye drops could interact hydrophobically with both the bulk lipid layer of the TFLL consisting of wax and cholesteryl esters and the monolayer of phospholipids at the aqueous interface of the TFLL. PL head group, the molecular weight of HA and calcium interactions are likely to be involved in PL–HA interactions [19–21]. The finding that HA interacts with the wax PP and mono-glyceride, PG, is relevant in that waxes and glycerides do not have a hydrophilic charged head group as PL do, so the lipid hydrocarbons chains can be involved in the interaction with HA as a head group is not necessary for HA–lipid interactions. However, head group interactions may be involved with HA–lipid interaction [19] and the current study shows PL bind much tighter with a lower maximum PL/HA ratio compared with wax/HA suggesting head group involvement. pSM initially decreased the intensity of the HA resonances much more than DPPC indicating that the hydrocarbon sphingosine back bone of pSM and the acyl linked hydrocarbon chains of DPPC influenced the interaction characteristics as the head group choline moieties are the same for both lipids. DPA without a choline head group but with the same acyl chains as DPPC both had a maximum binding ratio of 1:1, however, DPA initially decreased the intensity of the HA resonances much more than DPPC, indicating that head group binding could also be important. Despite the possible involvement of hydrocarbon chain–HA interactions, the interaction of PC with HA only minimally affects the enthalpy of the hydrocarbon phase transition [19], and both head group and hydrocarbon chain moieties influence HA–lipid interactions.

The PL mono-layer of the TFLL is composed of 72% PC and 10% SM, both shown in the current study to interact with HA [24, 44, 45]. One could speculate that strands of HA could lay flat on the posterior and anterior surface of the TFLL, ‘holding’ adjacent lipid molecules together, inhibiting tear breakup and increasing tear film stability. The amount of HA in tears is complicated by tear film clearance and whether HA–PL binding is reversible. Future studies involving native tear lipids, rheological studies and in vivo measurements are warranted.

HA–lipid binding could be important for topical skin-care products where evaporation of water from the product applied to the surface of the skin could lead to relatively high concentrations of HA. Our data show that the HA would be expected to bind to the sebatious wax and cholesteryl esters and subcutaneous PL.

## Conclusions

Physiological levels of HA, PC and sphingomyelin would result in 4% of the hydrophobic hydrogens of HA to be bound. HA–PL binding interactions could be important for therapeutic use of HA in eye drops to treat dry eye and to trap PL entering the VH to keep them from forming light scattering micelles. HA–lipid binding may also be relevant to the therapeutic effects of topical skin-care products. Both head group and hydrocarbon chain moieties influence HA–lipid interactions.

## Data Availability

Douglas Borchman, the contributing author, agrees to make all data available.

## Abbreviations

Cho: Cholesterol
DPPA: Dipalmitoyl phosphatidic acid
DPPC: Dipalmitoyl phosphatidylcholine
HA: Hyaluronic acid
NMR: Nuclear magnetic resonance
PG: Palmitoyl glyceride
PL: Phospholipid
PP: Palmitoyl palmitate
pSM: Palmitoyl sphingomyelin
TFLL: Tear film lipid layer
VH: Vitreous humor
